# High-Pressure Engineering for the Design of Ni^2+^-Based Phosphors

**DOI:** 10.1021/acsami.6c05592

**Published:** 2026-06-01

**Authors:** Mikołaj Kamiński, Yi-Ting Tsai, En-Pei Liu, Wei-Tin Chen, Mu-Huai Fang, Sebastian Mahlik

**Affiliations:** † Institute of Experimental Physics, Faculty of Mathematics, Physics and Informatics, 49646 University of Gdansk, Wita Stwosza 57, Gdansk 80-308, Poland; ‡ Research Center for Applied Sciences, 38017 Academia Sinica, Taipei 11529, Taiwan; § Department of Physics, 33561 National Taiwan University, Taipei 10617, Taiwan; ∥ Center for Condensed Matter Sciences and the Center of Atomic Initiative for New Materials, 33561 National Taiwan University, Taipei 10617, Taiwan; ⊥ Taiwan Consortium of Emergent Crystalline Materials, National Science and Technology Council, Taipei 10622, Taiwan

**Keywords:** Ni^2+^-doped phosphors, shortwave infrared emission, broadband emission, high-pressure engineering, high-pressure spectroscopy

## Abstract

High-pressure engineering provides a powerful strategy to activate and tailor the optical properties of transition-metal-based phosphors beyond the constraints of ambient-pressure crystal chemistry. Here, we demonstrate that pressure- and temperature-induced structural transformation of β-LiGaO_2_:Ni^2+^ to α-LiGaO_2_:Ni^2+^ stabilizes Ni^2+^ ions in an octahedral coordination environment, enabling intense broadband shortwave infrared (SWIR) emission arising from the spin-allowed ^3^T_2_ → ^3^A_2_ transition of the *d* ^8^ electronic configuration. In contrast, the as-prepared low-pressure β-LiGaO_2_:Ni^2+^ phase remains optically inactive. Temperature- and pressure-dependent photoluminescence, excitation, and lifetime measurements, combined with crystal-field analysis, reveal a weak octahedral crystal field, moderate electron–lattice coupling, and a pressure-dependent evolution of the Racah parameters in α-LiGaO_2_:Ni^2+^. Accounting for this evolution exposes a fundamental limitation of conventional Tanabe–Sugano diagrams, showing that the commonly anticipated ^1^E–^3^T_2_ crossover is shifted to much higher pressures or may become experimentally inaccessible. The unexpected increase of the Racah parameter *B* with pressure indicates enhanced localization of Ni^2+^ 3*d* electrons and reduced metal–ligand covalency. These results establish high-pressure engineering as an effective pathway for designing broadband Ni^2+^-based SWIR phosphors.

## Graphical abstract

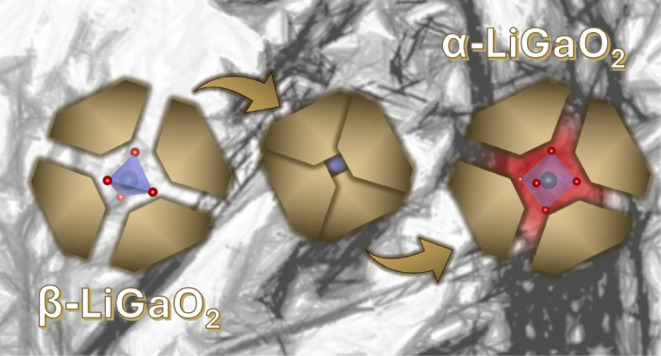

## Introduction

In recent years, near-infrared (NIR) optical technologies have garnered intense interest owing to their broad applicability in biomedical imaging and sensing, early cancer diagnostics, indoor agriculture, security monitoring, and food safety inspection.^1–5^ The rapid development of portable NIR spectroscopic platforms, including smartphones and wearable health devices, further underscores the growing demand for compact, efficient, and spectrally versatile NIR light sources. The NIR window is conventionally divided into NIR I (700–1000 nm) and NIR II (1000–1700 nm), with the latter offering clear advantages such as deeper tissue penetration, suppressed photon scattering and absorption, higher signal-to-noise ratios, and superior spatial resolution.^6–8^ Despite these advantages, realizing high-performance NIR II emitters that simultaneously deliver ultrabroadband emission (full width at half-maximum >200 nm) and high radiative efficiency remains a formidable challenge. Conventional NIR sources, including tungsten-halogen lamps and diode lasers, offer high output power but are limited by excessive energy consumption, bulky architectures, high cost, or intrinsically narrow emission bandwidths. In this context, phosphor-converted light-emitting diodes (pc-LEDs) have emerged as a compelling alternative, combining broadband emission, high brightness, low coherence, compactness, and low power consumption with scalable and cost-effective fabrication.^9,10^

A central element of pcLEDs is the phosphor activator. Among them, Ni^2+^ with its 3*d* ^8^ electronic configuration, emerges as particularly promising for a new generation of ultrabroadband NIR-II phosphors. Its spin-allowed ^3^T_2_ → ^3^A_2_ transition produces broadband NIR-II emission spanning 1000–1700 nm, with a fwhm values exceeding 300 nm^11,12^ and reaching up to ∼340 nm,^13^ making Ni^2+^-doped phosphors highly attractive. Notable examples include MgO, MgAl_2_O_4_, MgGa_2_O_4_, Sr_2_AlTaO_6_, and Y_3_Al_2_Ga_3_O_12_.^14–18^ While many studies have emphasized the practical application aspects of Ni^2+^ emission, relatively few have systematically explored the optical properties and fundamental physical processes governing it.^19–21^ Such insights are essential for establishing Ni^2+^ as a central component in NIR-II pc-LEDs.

To optimize or tune the luminescent properties of Ni-doped phosphors, the solid-solution approach has emerged as the most straightforward and practical strategy. It allows control over structural rigidity, thereby enhancing the thermal stability of Ni^2+^-doped materials,^22^ or can be used to modulate the crystal field strength, as demonstrated in Ni-doped Li­(Ga–Al)_5_O_8_ and Mg­(Al–Ga–Sn)_2_O_4_,^23,24^ resulting in fine-tuning of their spectral properties. Another strategy involves optimizing the synthesis process through various techniques, such as hydrothermal, sol–gel, or water-assisted solid-state reactions, the addition of fluxes, such as H_3_BO_3_, during sintering, or postsynthesis treatments.^25,26^ Nevertheless, these approaches remain constrained mainly by the limited structural diversity accessible through compositional tuning alone, underscoring the urgent need for alternative design strategies to unlock new luminescent behaviors.

Here, we introduce a pressure and temperature induced crystallographic engineering strategy for designing new Ni-doped phosphors. Ni^2+^ ions were doped into the β-LiGaO_2_ structure, which is entirely composed of [LiO_4_] and [GaO_4_] tetrahedra and is incapable of emitting Ni^2+^ luminescence. Our strategy successfully transformed β-LiGaO_2_:Ni^2+^ into α-LiGaO_2_:Ni^2+^, enabling broadband NIR-II emission centered around 1500 nm. The α-LiGaO_2_:Ni^2+^ exhibits strong luminescence associated with octahedrally coordinated Ni^2+^, which has been thoroughly examined using methods such as synchrotron X-ray absorption spectroscopy, and temperature- and pressure-dependent analyses. This work not only introduces a previously unexplored NIR-emitting phosphor system but also provides fundamental insights into pressure-driven structural and electronic evolution, establishing pressure engineering as a powerful paradigm for phosphor design.

## Results and Discussion

### Structural Properties

The structural characteristics of LiGa_0.99_Ni_0.01_O_2_ synthesized under high-pressure (HP-LGON) and ambient pressure (AP-LGON) were analyzed using high-resolution synchrotron X-ray diffraction (XRD) patterns, as illustrated in Figure 1a. The standard diffraction patterns for HP-LGON and AP-LGON were obtained from the Crystallographic Information Framework (CIF), with reference numbers ICSD-28388 and ICSD-14987, respectively.^27,28^ To provide detailed insight into the lattice parameters and crystallite size, Rietveld refinement was performed to determine the crystal structures and quantify the phase compositions of HP-LGON and AP-LGON, as illustrated in Figure 1b and 1c, respectively. The refined structural parameters, including lattice constants, atomic positions, site occupancies, and atomic displacement parameters, are summarized in Tables S1 and S2. HP-LGON adopts the α-LiGaO_2_ structural framework, belonging to the trigonal crystal system with space group *R*3̅*m*. In this structure, both Li^+^ and Ga^3+^ are coordinated by six O^2–^ ions, forming octahedral units [LiO_6_] and [GaO_6_], as depicted in Figure 1d. The HP-LGON structure consists of a three-dimensional network of edge-sharing [LiO_6_] and [GaO_6_] octahedra.^29^ Within this framework, the six Li–O and Ga–O bonds in each octahedron are of equal length, with the average Li–O bond being slightly longer than the Ga–O bond. Repeated XRD measurements confirmed that HP-LGON retained the pure high-pressure α-phase without detectable impurity formation or α → β phase transition after nearly two years under ambient conditions. In contrast, AP-LGON crystallizes in the β-LiGaO_2_ structure, which belongs to the orthorhombic crystal system, space group *Pna*2_1_. In this phase, both Li^+^ and Ga^3+^ ions are coordinated by four O^2–^ ions to form tetrahedral units [LiO_4_] and [GaO_4_], which share vertices, as shown in Figure 1e. These tetrahedra exhibit two distinct bond lengths (Li1–O1/Li1–O2 and Ga1–O1/Ga1–O2), reflecting subtle structural distortions inherent to the orthorhombic β-phase.^30^

**Figure 1:**
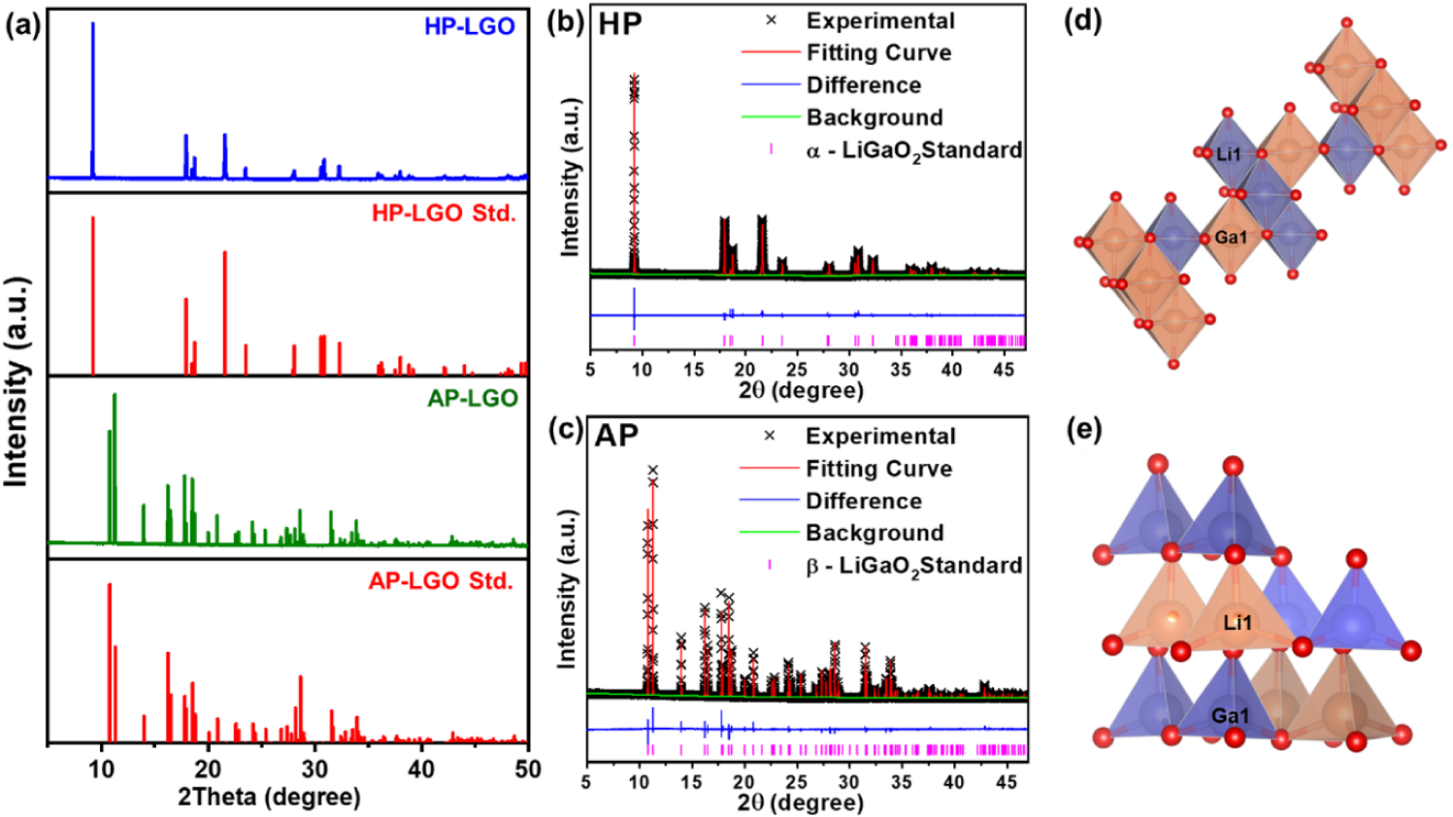
(a) High-resolution synchrotron XRD patterns of the AP-LGON and HP-LGON. Rietveld refinement of (b) HP-LGON and (c) AP-LGON. Crystal structure of (d) α-LiGaO_2_ (HP-LGON) and (e) β-LiGaO_2_ (AP-LGON).

### Morphology and X-ray Absorption Analyses (XAS)

Optical microscopy (OM) and scanning electron microscopy (SEM) were employed to characterize the particle morphology of the samples. Figure 2a and 2b show OM images of HP-LGON acquired at magnifications of 150× and 500×, respectively. The HP-LGON powder exhibits relatively large, well-defined particles with a high degree of crystallinity. A subtle green sheen is observed on the salt particles, attributable to Ni^2+^ absorption, and the HP-LGON crystals display high optical transparency.

**Figure 2:**
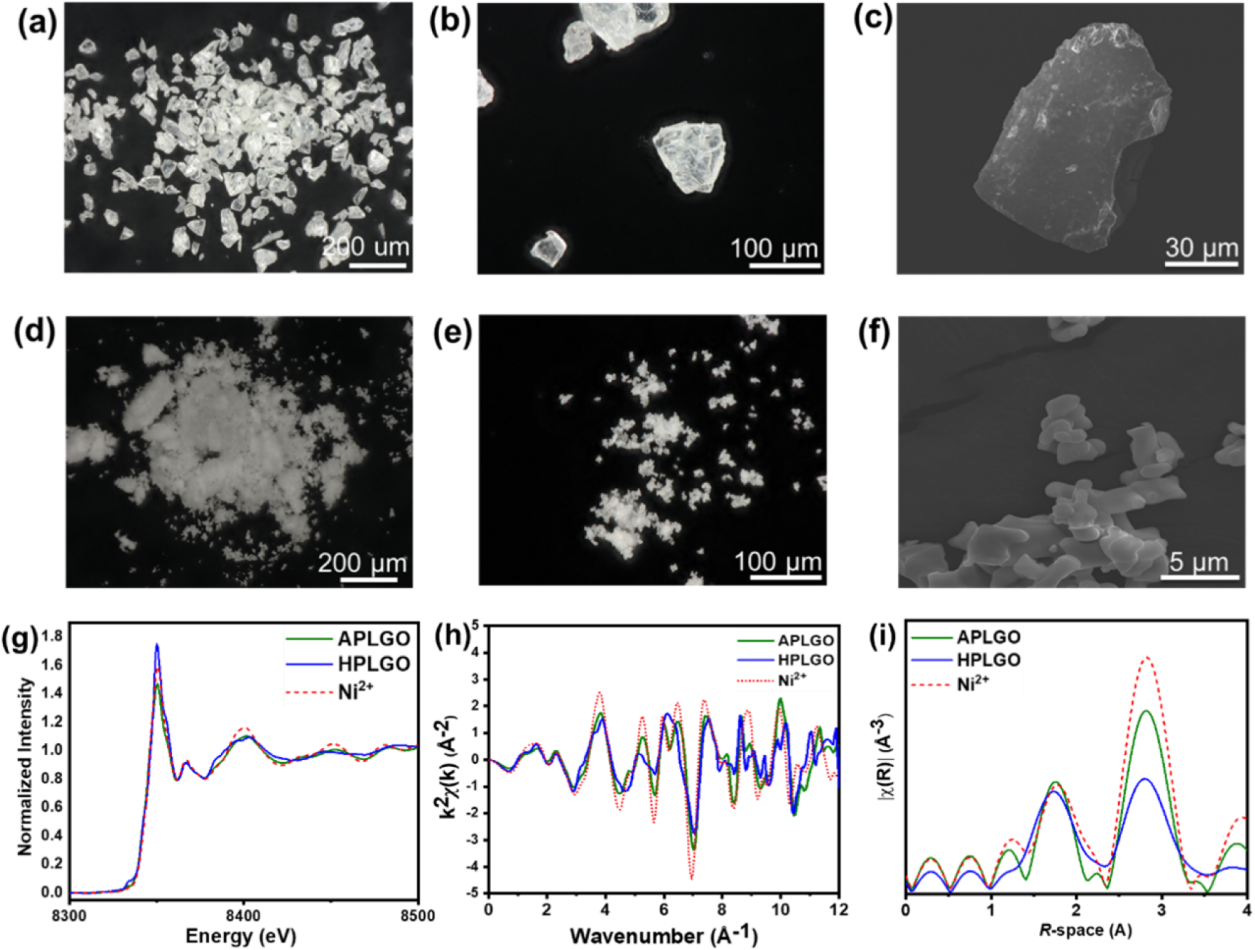
Optical microscopy (OM) images of (a, b) HP-LGON acquired at magnifications of 150× and 500×, respectively, and the corresponding SEM image (c) recorded at 2,000×. OM images of (d, e) AP-LGON acquired at magnifications of 150× and 500×, respectively, and the corresponding SEM image (f) recorded at 13,000×. (g) Ni *K*-edge XANES spectra of AP-LGON and HP-LGON compared with a Ni^2+^ standard. (h) *k* ^2^-weighted Fourier transforms of the Ni *K*-edge EXAFS spectra. (i) *R*-space EXAFS spectra at the Ni *K*-edge.

The enhanced crystallinity and large particle size are further confirmed by the SEM image in Figure 2c, obtained at a magnification of 2,000×, which reveals flat crystal surfaces and particle dimensions approaching ∼100 μm, consistent with the OM observations. In contrast, AP-LGON shows markedly different morphological characteristics. The OM images (Figure 2d and 2e) indicate that the AP-LGON powder appears white and consists of much smaller particles, making individual grains difficult to resolve even at higher magnification. This observation is clarified by the SEM image in Figure 2f, acquired at a magnification of 13,000×, which reveals particle sizes in the range of 1–5 μm. Owing to the substantial disparity in particle size between HP-LGON and AP-LGON, direct comparison at identical SEM magnifications was not feasible. Overall, the OM and SEM results clearly demonstrate pronounced differences in particle morphology and crystallinity between LGO crystals synthesized under high-pressure and ambient-pressure conditions, as revealed by comprehensive OM and SEM characterization. X-ray absorption near-edge structure (XANES) analysis at the Ni *K*-edge is a powerful tool for probing changes in oxidation state. The Ni *K*-edge XANES spectra of HP-LGON and AP-LGON are presented in Figure 2g. Both spectra closely match the standard reference for Ni^2+^ (NiO), confirming that nickel predominantly exists in the +2 oxidation state.

To further elucidate the local environment of Ni^2+^, extended X-ray absorption fine structure (EXAFS) analysis was performed. The Ni *K*-edge *k* ^2^-weighted Fourier transforms of EXAFS for AP-LGON and HP-LGON are shown in Figure 2h The AP-LGON structure consists exclusively of tetrahedral units and does not contain any octahedral polyhedral units. Besides, the Ni^2+^ precursors in AP-LGON and the Ni^2+^ standard in EXAFS are both NiO. Accordingly, the *k* ^2^-weighted Fourier transforms of EXAFS results, which the Ni oscillation pattern in AP-LGON closely resembles the one in NiO precursor, may imply that the Ni is not successfully incorporated into the AP-LGO framework. Instead, Ni is more likely to exist as a separate phase. By contrast, although both HP-LGON and NiO exhibit octahedral coordination, their oscillation patterns differ considerably. This discrepancy likely arises from local distortions associated with Ni^2+^ substitution at both Li and Ga sites to maintain charge neutrality.

The Ni *K*-edge *R*-space EXAFS spectra for AP-LGON and HP-LGON are displayed in Figure 2i. The first coordination shell of Ni–O interactions in AP-LGON shows a radial distribution that closely resembles that of the Ni^2+^ standard. Similarly, the *R*-space signal in HP-LGON appears at a comparable distance, consistent with octahedral coordination. In the HP-LGON structure, both [LiO_6_] and [GaO_6_] are octahedral sites. Considering the ionic radii of Ga^3+^ (0.62 Å, CN = 6), Li^+^ (0.76 Å, CN = 6), and Ni^2+^ (0.69 Å, CN = 6), substitution of Ni^2+^ at both cation sites is expected to induce expansion of the Ga site and contraction of the Li site. This local structural adjustment yields a balanced configuration, leading to only subtle variations in the *R*-space spectra.

Overall, the EXAFS results, when combined with the XRD analysis, provide a comprehensive understanding of the local coordination and site occupancy of Ni^2+^ in LGON synthesized under different pressure conditions. This result also demonstrates the critical role of EXAFS in determining whether the transition-metal ions are incorporated into the host structure, especially for those with low doping concentration.

### Photoluminescence Properties

The AP-LGON sample exhibited no detectable Ni^2+^ luminescence, consistent with the XANES and EXAFS results, which indicated that Ni^2+^ ions are not incorporated into tetrahedral sites. In contrast, the HP-LGON sample, in which Ni^2+^ occupies octahedral coordination sites, displays strong broadband luminescence in the shortwave infrared (SWIR) region. Consequently, subsequent detailed luminescence investigations were focused on the HP-LGON sample. The photoluminescence excitation (PLE) spectrum of HP-LGON recorded at room temperature (RT) is shown in the inset of Figure 3a. The PLE spectrum, measured in the 320–1000 nm range, exhibits two dominant broad bands. The first band, centered at 420 nm, corresponds to the ^3^A_2_ → ^3^T_1_(^3^P) spin-allowed transition. Within this band, a weaker high-energy shoulder observed in the 450 and 500 nm is attributed to the spin-forbidden ^3^A_2_ → ^1^T_2_ transition.^31,32^

**Figure 3:**
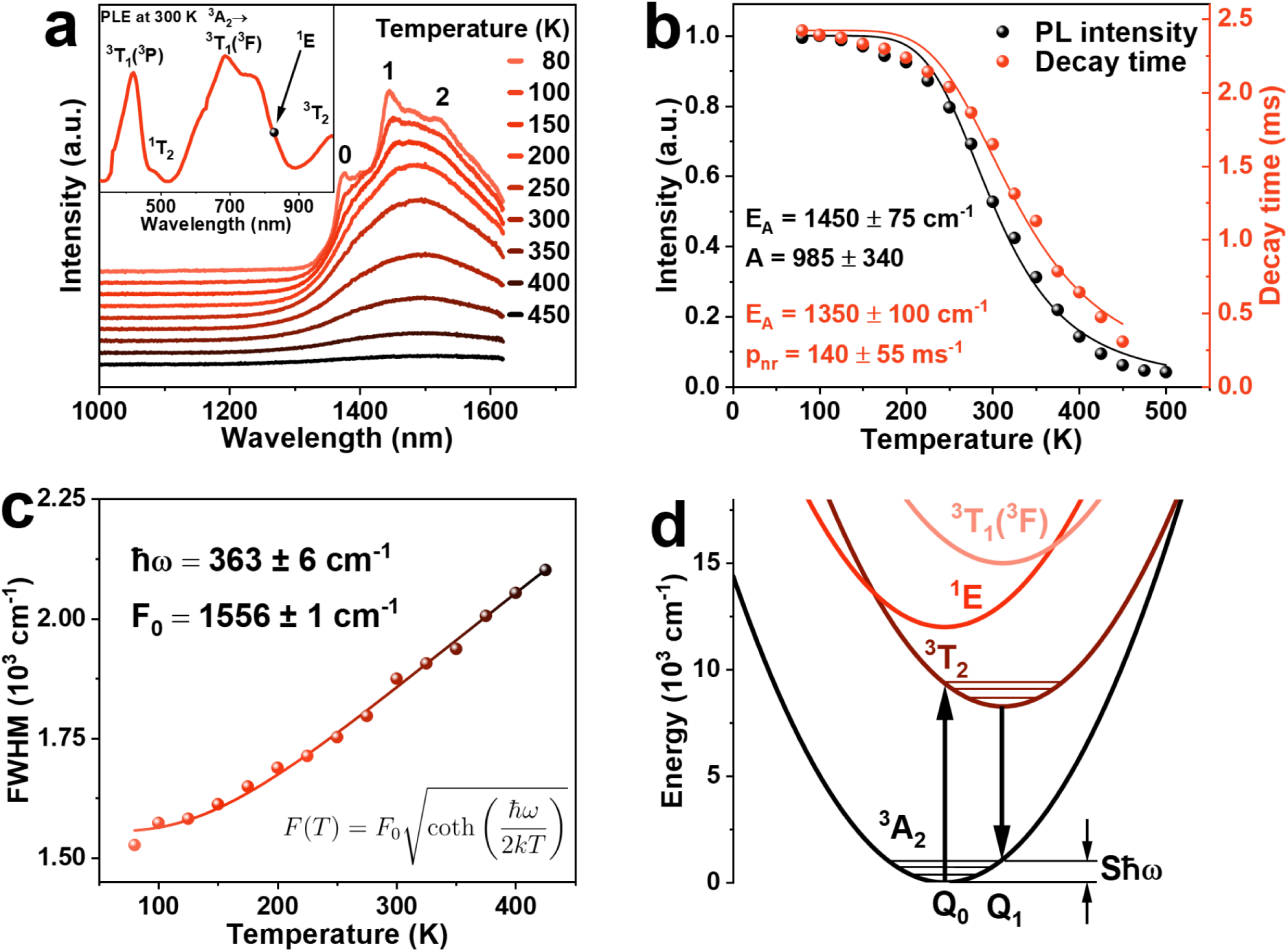
Temperature dependence of (a) photoluminescence spectra of HP-LGON under 410 nm excitation; the inset shows the room-temperature photoluminescence excitation spectrum recorded for the Ni^2+^ emission, (b) total integrated intensities and decay times, (c) full widths at half-maximum (fwhm). (d) Configurational coordinate diagram corresponding to HP-LGON.

The second band extends from 550 to 850 nm and displays a more complex structure, as previously reported for octahedrally coordinated (*d* ^8^) Ni^2+^ ions.^20,33^ This band is primarily attributed to the ^3^A_2_ → ^3^T_1_(^3^F) transition. The observed asymmetry and broadening of this band can arise from low-symmetry distortions of the NiO_6_ octahedra in the HP-LGON lattice, as well as from a weak contribution of the spin-forbidden ^3^A_2_ → ^1^E transition on the low-energy side. Consequently, to determine the energy of the ^3^A_2_ → ^3^T_1_(^3^F) transition, a two-Gaussian fit was applied, and the energy of the dominant Gaussian component was selected (see Figure S1a). Lastly, the third band, which appears above 900 nm, is attributed to the ^3^A_2_ → ^3^T_2_ transition. Owing to the spectral limitations of our experimental setup, this band is only partially observable both at room temperature and under cryogenic conditions. The photoluminescence excitation spectrum of HP-LGON recorded at cryogenic temperature is shown in Figure S1b.

Obtaining the transition energies allows us to determine the crystal field splitting parameter *Dq* and the Racah parameters *B* and *C*. Equations 1 and 2 illustrate how *Dq* and *B* can be calculated using the energies of the ^3^A_2_ → ^3^T_1_(^3^P) and ^3^A_2_ → ^3^T_1_(^3^F) transitions. The Racah parameter *C*, which predominantly governs the energy of spin-forbidden transitions in the *d* ^8^ configuration, is subsequently determined from the energy of the ^3^A_2_ → ^1^T_2_ transition, as given in eq 3. The derivations of eqs 1 and 2, as well as the methodology for eq 3, are provided in the refs 34,35 .

1 $$340Dq^{2}-18Dq\left(E_{1}+E_{2}\right)+E_{1}E_{2}=0$$

2 $$B=\frac{E_{1}+E_{2}-30Dq}{15}$$

3 $$C=\frac{E_{3}}{2}-\frac{17B+30Dq-\sqrt{49B^{2}+20BDq+100Dq^{2}}}{4}$$

where *E*_1_, *E*_2_, and *E*_3_ are equal to energy of ^3^A_2_ → ^3^T_1_(^3^P), ^3^A_2_ → ^3^T_1_(^3^F), and ^3^A_2_ → ^1^T_2_ transitions, respectively. The calculated parameters are *Dq* = 896 cm^–1^, *B* = 767 cm^–1^, and *C* = 3153 cm^–1^. The resulting ratio *Dq*/*B* = 1.17 indicates that Ni^2+^ ions in HP-LGON experience a weak octahedral crystal field.^36^ Determination of the *Dq*, *B*, and *C* parameters allows the calculation of all energy levels for the *d* ^8^ electronic configuration, as these levels can be expressed as linear combinations of the above parameters.^37^ Based on this analysis, the energy of the ^1^E state relative to the ^3^A_2_ ground state was obtained to be equal to 12070 cm^–1^, represented by the black sphere in the inset of Figure 3a. Finally, the calculated energy of the ^3^T_2_ state relative to the ground state is expressed as 10 *Dq* = 8960 cm^–1^.

To analyze the electron–lattice interactions, the spectra are represented as lineshapes *L­(ν)* as a function of wavenumber ν, using *L­(ν) ∝ I­(ν) ν* ^–3^ for emission and *L­(ν) ∝ I­(ν) ν* ^–1^ for excitation spectra, where *I­(ν)* denotes the emission or excitation intensity.^38^ Figure 3a presents the temperature-dependent photoluminescence (PL) spectra excited at 410 nm over the 80–450 K range. At low temperatures, the spectra exhibit a well-resolved phonon structure (Stokes sidebands), with successive phonon replicas labeled 0, 1, and 2. The 0 replica corresponds to the zero-phonon line (ZPL) at 7263 cm^–1^ (1377 nm), while the energy separations between the 0–1 and 1–2 replicas are 350 cm^–1^ and 353 cm^–1^, respectively. The phonon energy of ∼ 350 cm^–1^ inferred from the spacing of the Stokes replicas is consistent with lattice modes involving deformations/rotations of MO_6_ octahedra in the α-LiGaO_2_-type framework *R*3̅*m*, where Raman bands near ∼352 cm^–1^ have been reported for this phase.^39^ This suggests that the observed vibronic progression is predominantly coupled to octahedral lattice vibrations rather than high-frequency internal M–O stretching modes. A similar results has been shown in the study of HPLGO:Cr, where pressure-dependent Raman spectra are additionally presented.^30^ The 300 K PL spectrum of HP-LGON exhibits a broad SWIR emission band originating from the spin-allowed ^3^T_2_ → ^3^A_2_ transition of Ni^2+^ in an octahedral *d* ^8^ configuration. The emission extends from approximately 1250 nm to beyond the detection limit of the experimental setup at 1700 nm, with a maximum intensity near 1500 nm and a full width at half-maximum (fwhm) of 278 nm (1875 cm^–1^). Such a broad emission bandwidth is characteristic of Ni^2+^-activated phosphors with octahedral coordination and reflects strong electron–phonon coupling associated with the ^3^T_2_ excited state. Compared with other Ni^2+^-doped oxide and garnet hosts reported in the literature, the emission maximum and bandwidth of HP-LGON fall well within the typical SWIR range, highlighting its potential as a broadband SWIR-emitting material. The temperature dependence of decay profiles under 420 nm excitation is shown in Figure S2a. The decay dynamics become progressively faster with increasing temperature; at low temperatures, the profiles are well described by a nearly single-exponential behavior, whereas at higher temperatures they evolve into increasingly multiexponential decays. To account for this behavior, the average decay times of Ni^2+^ luminescence were calculated using (eq 4)­

4 $$\tau_{av}=\frac{\int I\left(t\right)tdt}{\int I\left(t\right)dt}$$

Where *I­(t)* is emission intensity at time *t*. Figure 3b presents the temperature-dependent average decay times calculated using (eq 4) (orange spheres), together with the corresponding integrated total PL intensities (black spheres). Both quantities exhibit similar temperature dependences, characterized by a gradual decrease up to approximately 250 K, followed by a rapid drop at higher temperatures, indicating that the same thermally activated process governs the luminescence quenching. Accordingly, the temperature evolution of the total integrated emission intensity and the average decay time was further analyzed using (eqs 5 and 6), respectively:

5 $$I\left(T\right)=\frac{I_{0}}{1+A\exp\left(-\frac{E_{A}}{kT}\right)}$$

6 $$\tau \left(T\right)=\frac{1}{\tau_{0}^{-1}+p_{nr}\exp\left(-\frac{E_{A}}{kT}\right)}$$

where *I*_0_ is the initial intensity of the emission, *A* is the relative probability of the nonradiative deexcitation processes, *E*_*A*_ is an activation energy, τ_0_ is an initial decay time, and *p*_*nr*_ is a nonradiative parameter. The parameters are summarized in Table S3. Furthermore, the temperature dependence of the fwhm is presented in Figure 3c. The fwhm increases monotonically with rising temperature due to the increasing thermal population of phonons interacting with the electronic states of Ni^2+^. The temperature evolution of the emission bandwidth was analyzed using the hyperbolic cotangent law (eq 7), which explicitly accounts for phonon occupation effects at a fixed electron–phonon coupling strength,^40^

7 $$F\left(T\right)=F_{0}\sqrt{\coth\left(\frac{\hbar \omega }{2kT}\right)}$$

where *ℏω* is the energy of the effective phonon, and *F*_0_ is an arbitrary fwhm at 0 K and can also be expressed as *F*_0_ = 2.36*ℏωS* ^1/2^, where *S* is a Huang–Rhys parameter. From the fitting, we obtained *F*_0_ = 1556 ± 1 cm^–1^ and *ℏω* = 364 ± 6 cm^–1^, which closely match those derived from the low-temperature emission spectrum. Using the above expression for *F*_0_ yields *S* = 3.28 ± 0.11. A quantitative measure of the electron–lattice coupling is provided by the Stokes shift, defined as *2Sℏω,* which represents the energy separation between the maxima of the photoluminescence excitation (PLE) and photoluminescence (PL) spectra. From the present analysis, *2Sℏω* was determined to be 2389 ± 120 cm^1^. Within this framework, the energy of the ^3^T_2_ excited state relative to the ground state is predicted to be 8890 cm^–1^, which is in close agreement with the value obtained independently from the crystal-field parameter analysis (8960 cm^–1^).

Figure 3d presents the configurational coordinate diagram for HP-LGON, constructed using the experimentally derived *Sℏω* values for the ground and excited states obtained from the analysis above. The diagram depicts the potential energy curves of the ^3^A_2_ ground state and the excited ^3^T_2_, ^1^E, and ^3^T_1_(^3^P) states of Ni^2+^ as functions of the configurational coordinate *Q*. The displacement between the ground and excited state equilibrium positions along *Q* arises from electron–lattice interactions. Within the harmonic approximation, the lattice relaxation energy *Sℏω* is proportional to the square of the configurational displacement, *Sℏω ∝ (Q*_1_ *– Q*_0_)^2^, where *Q*_0_ and *Q*_1_ denote the equilibrium coordinates of the ground and the lowest excited state ^3^T_2_, respectively. Notably, under ambient conditions, the crossover point between the ^3^T_2_ and ^3^A_2_ potential energy curves, typically associated with thermally activated nonradiative quenching, occurs at energies significantly higher than those inferred from the temperature-dependent luminescence measurements, suggesting that classical crossover-mediated relaxation is unlikely to dominate. In this case, the observed luminescence quenching may instead originate from thermally activated nonradiative relaxation via defect-related or trap states within the band gap, or from local energy transfer to quenching centers, providing efficient nonradiative pathways.

To place the temperature-dependent luminescence results in a broader context, a comparison with previously reported Ni^2+^ and Cr^3+^/Ni^2+^activated oxide phosphors is provided in Table S4. The present material exhibits comparable performance in terms of emission maxima, fwhm, and activation energy. Notably, further improvements may be anticipated upon additional Cr^3+^ doping, as suggested by related systems reported in the literature.

### Pressure Effects on Electronic Structure and Luminescence

The pressure-dependent photoluminescence excitation (PLE) spectra of Ni^2+^ emission in HP-LGON measured up to 17.6 GPa are shown in Figure 4a. With increasing pressure, all excitation bands systematically shift toward higher energies, in agreement with crystal-field theory. An increase in mechanical pressure enhances the crystal-field strength; for the *d^8^* electronic configuration, this results in an upward shift of the corresponding electronic energy levels. In addition, the band associated with the ^3^A_2_ → ^3^T_1_(^3^F) transition, which exhibits a complex structure under ambient conditions, progressively loses its low-energy component as pressure increases, indicating pressure-induced modification of the local crystal-field environment and reduced low-symmetry contributions. Figure 4b shows the pressure-dependent photoluminescence (PL) spectra of HP-LGON recorded under 658 nm excitation up to 21.6 GPa. The spectra exhibit a systematic shift of the broadband, spin-allowed ^3^T_2_ → ^3^A_2_ emission toward higher energies with increasing pressure, while the full width at half-maximum (fwhm) remains nearly constant over the investigated pressure range. The corresponding pressure-dependent transition energies are summarized in Figure 4c. The energy of the ^3^A_2_ → ^3^T_1_(^3^F) transition was extracted using a double-Gaussian fitting procedure, analogous to that employed for the room-temperature PLE spectra. Notably, the pressure-induced energy shifts are nonlinear. All transitions display a more rapid increase in energy up to approximately 9 GPa, followed by a gradual reduction in slope at higher pressures. Using the pressure dependences of the transition energies and applying (eqs 1–3), the pressure evolution of the crystal-field splitting parameter *Dq* and the Racah parameters *B* and *C* was determined. The results are shown in Figure 4d. From these data, the corresponding pressure coefficients were extracted as d*Dq*/d*P* = 4.5 ± 0.9 cm^–1^/GPa, d*B*/d*P* = 6.8 ± 0.8 cm^–1^/GPa, and d*C*/d*P* = 6.0 ± 2.5 cm^1^/GPa. The observed increase of the Racah parameter B with pressure may indicate enhanced localization of the Ni^2+^ 3d electrons and a reduction of metal–ligand covalency, suggesting that compression predominantly strengthens electrostatic crystal-field interactions rather than orbital hybridization. This behavior likely reflects a combination of octahedral stiffening, a reduced nephelauxetic effect, and pressure-induced suppression of local structural distortions, including decreased asymmetry and a narrower distribution of Ni–O bond lengths.

**Figure 4:**
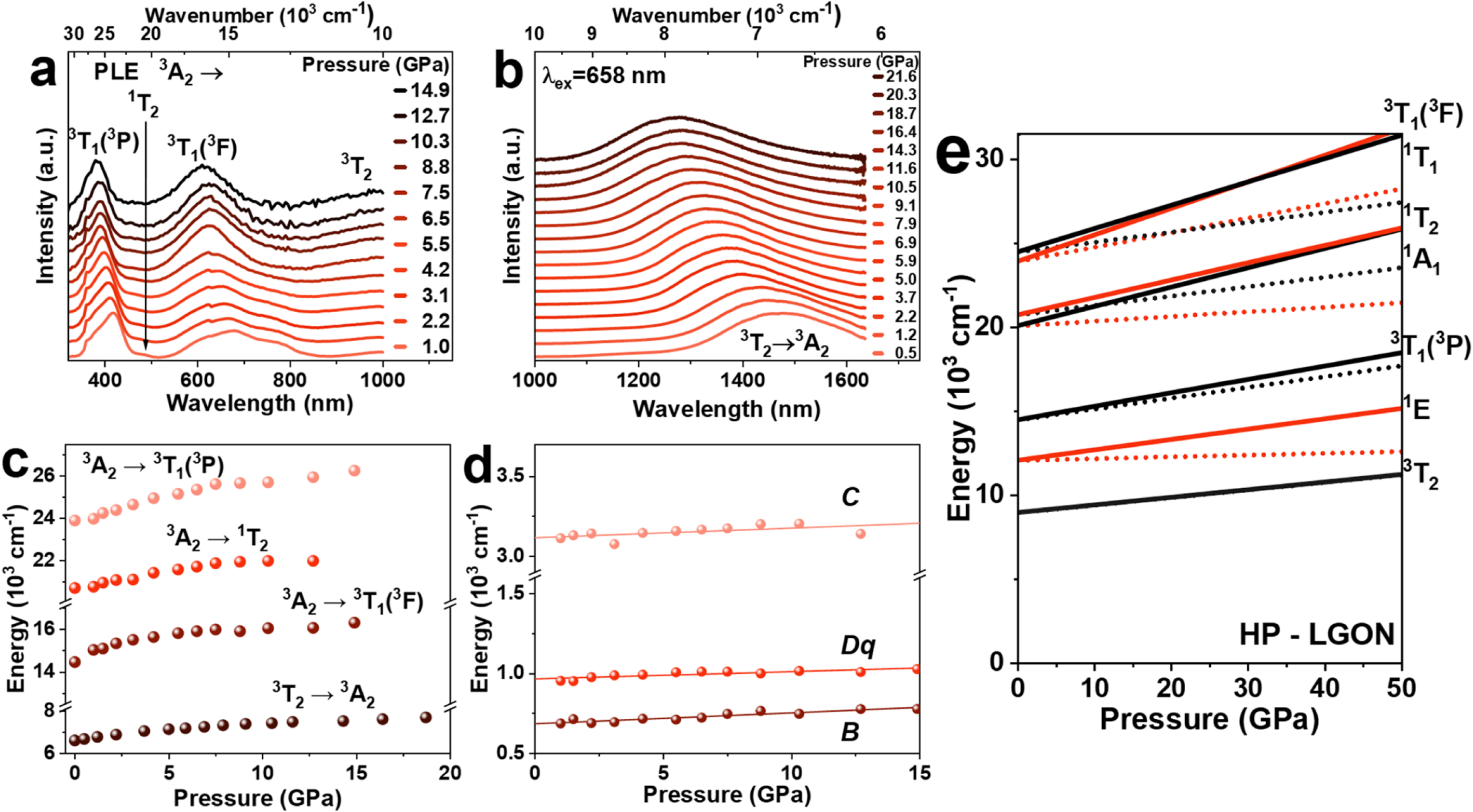
HP-LGON pressure-dependent (a) photoluminescence excitation spectra of Ni^2+^ luminescence, (b) photoluminescence spectra upon 658 nm excitation, (c) positions of excitation and emission bands, (d) calculated crystal field strength *Dq*, and Racah parameters *B* and *C*, (e) energy structure diagram of Ni^2+^ ion in *d^8^* configuration.

By determining the crystal field strength (*Dq*) and the Racah parameters (*B* and *C*) as functions of applied pressure, a comprehensive energy-level diagram for the Ni^2+^ ion in the *d* ^8^ configuration can be constructed. Each energy level in this configuration can be expressed as a combination of *Dq*, *B*, and *C.* ^37^ Experimental determination of the pressure dependences of these parameters enables evaluation of their respective pressure coefficients. Extrapolation of these trends allows calculation of the complete set of *d* ^8^ energy levels as functions of pressure. The resulting pressure-dependent energy levels, extrapolated over the 0–50 GPa range, are presented in Figure 4e. Traditional Tanabe–Sugano (T–S) diagrams assume constant values for the Racah parameters *B* and *C*, which are shown in Figure 4e as dotted lines. However, experimental results clearly demonstrate that both *B* and *C* vary with applied pressure. Incorporating these pressure dependences yields a more realistic energy-level scheme, shown by the continuous lines in Figure 4e.

A key, nontrivial outcome of this analysis for the optically active states is that it reveals a fundamental limitation of conventional T–S diagrams when applied under high-pressure conditions. While standard T–S diagrams predict a crossover between the ^1^E and ^3^T_2_ states at moderate crystal-field strengths, our pressure-dependent analysis shows that this crossover shifts to significantly higher pressures or may even become inaccessible within the experimentally relevant range. As a result, direct application of conventional T–S diagrams to high-pressure systems can lead to qualitatively misleading conclusions regarding the ordering and crossing of excited electronic states.

In contrast, the identical pressure evolution of the ^3^T_2_ state in both the conventional T–S diagram and the pressure-dependent model arises from its strictly linear dependence on the crystal-field strength *Dq*, given by E­(^3^A_2_ → ^3^T_2_) = 10*Dq*. Because *Dq* increases approximately linearly with pressure, the energy of the ^3^T_2_ state exhibits a correspondingly linear pressure dependence, independent of variations in the Racah parameters.

The decay profiles of Ni^2+^ luminescence in HP-LGON under 658 nm excitation at pressures up to 27.3 GPa are shown in Figure S2b. With increasing pressure, the decay curves become progressively faster and deviate more strongly from single-exponential behavior. The pressure-dependent average decay times were calculated using (eq 4) and are presented in Figure S2c. Upon initial compression, the average decay time increases slightly from 1.65 to 1.71 ms at 3 GPa, which can be attributed to the pressure-induced blueshift of the emission band. At higher pressures, the decay time gradually decreases, reaching 0.59 ms at 27 GPa, reflecting enhanced nonradiative relaxation and pressure-induced luminescence quenching. Notably, this behavior does not indicate an increase in the involvement of the ^1^E state, as its energetic separation from the ^3^T_2_ remains nearly pressure-independent. Moreover, lattice stiffening under compression suggests that tunneling-assisted relaxation is unlikely to be enhanced at high pressure. Instead, the observed quenching may be associated with pressure-enhanced depopulation of the Ni^2+^ excited state, driven by a progressive reduction of the energy separation between the ^3^T_2_ level and defect-related or trap states within the band gap, as well as by increasing energy transfer to quenching centers.

Additionally, the bulk modulus *B*_0_ ([Disp-formula eq8]) was calculated based on the pressure-dependent spectroscopic data using eq 9 ^41^

8 $$B_{0}=-V\frac{dP}{dV}$$

9 $$\frac{dDq}{dP}=-nDq\frac{1}{R}\frac{dR}{dP}=Dq\frac{nK}{3B_{0}}$$

Where, *K* is a parameter that reflects how pressure affects the local environment of the central ion: *K* = 1 indicates equal compression to the bulk lattice, *K* < 1 less compression, and *K* > 1 greater compression. The parameter *n* describes the radial dependence of the crystal-field strength (*Dq* ∝ *R* ^–n^) and is typically expected to be close to *n* = 5. Here, *R* denotes the average metal–ligand distance, and *V* is the unit-cell volume.

Based on the obtained data, and assuming that the local compressibility around the Ni^2+^ ion is equal to the lattice compressibility (*K* = 1), the bulk modulus was determined to be 330 ± 75 GPa. It should be noted that, according to the literature, for transition metal ions in various materials, the product *nK* is typically smaller than 5,^41,42^ implying that the local coordination environment is generally less compressible than the bulk lattice. Consequently, the *B*_0_ value reported here should be regarded as an upper limit. To the best of our knowledge, no experimental bulk modulus has been reported for LiGaO_2_ in the trigonal crystal system with space group *R*3̅*m*. For comparison, studies on α-LiGaO_2_ in the hexagonal crystal system report bulk modulus values of 142.29 GPa^43^ and 166 GPa.^44^ The apparently higher stiffness of HP-LGON may reflect the denser, octahedrally coordinated framework of the high-pressure phase, which is expected to be less compressible than the tetrahedrally coordinated structure of α-LiGaO_2_.

## Conclusions

We demonstrate that high-pressure engineering is an effective strategy for designing Ni^2+^-based phosphors, enabling control over local coordination environments, electronic structure, and optical properties. The pressure-induced transformation of AP-LGON into the HP-LGON phase stabilizes Ni^2+^ in an octahedral environment, activating intense broadband SWIR emission from the spin-allowed ^3^T_2_ → ^3^A_2_ transition. Pressure- and temperature-dependent spectroscopic studies combined with crystal-field analysis reveal a weak crystal field and moderate electron–lattice coupling, consistent with the observed broadband emission. Importantly, experimental determination of the pressure dependence of *Dq*, *B*, and *C* exposes a key limitation of conventional Tanabe–Sugano diagrams, allowing the Racah parameters to vary with pressure shifts the predicted ^1^E–^3^T_2_ crossover to much higher pressures or renders it inaccessible, demonstrating that standard Tanabe–Sugano diagrams can be qualitatively misleading under compression. The unexpected increase of the Racah parameter *B* with pressure suggests enhanced localization of Ni^2+^ 3*d* electrons and reduced metal–ligand covalency, indicating that compression predominantly strengthens electrostatic crystal-field interactions. These results demonstrate that pressure-driven control of coordination symmetry and crystal-field strength can stabilize optically active Ni^2+^ centers and tune their electronic structure, providing concrete design rules for SWIR-emitting phosphors based on experimentally validated pressure-dependent crystal-field parameters.

## Experimental Section

This study employed LiGaO_2_ doped with Ni^2+^ samples with nominal compositions LiGa_0.99_Ni_0.01_O_2_ were prepared using solid-state-derived precursor mixtures. Stoichiometric amounts of Li_2_CO_3_ (99.99%, Thermo), Ga_2_O_3_ (99.99%, Gredmann), and NiO (99.99%, LTS Chemical Inc.) were accurately weighed according to the desired molar ratios and thoroughly mixed by grinding in an agate mortar to ensure compositional homogeneity before subsequent processing.

### Synthesis of AP-LGO Powder

Ambient-pressure LiGa_0.99_Ni_0.01_O_2_ (denoted as AP-LGON) was synthesized by transferring the homogenized precursor mixture into alumina crucibles and calcining it in a tube furnace under an air atmosphere. The sample was heated to 900 °C and held for 2 h, then heated to 1000 °C for an additional 2 h. Heating and cooling rates were maintained at 5 °C min^–1^. After cooling to room temperature, the product was removed from the furnace and ground into a fine powder.

### Synthesis of HP-LGO Powder

High-pressure LiGa_0.99_Ni_0.01_O_2_ (denoted as HP-LGON) was obtained by subjecting the AP-LGO powder to high-pressure treatment. The precursor powder was sealed in a platinum capsule and processed in a cubic-anvil-type high-pressure apparatus at 4 GPa and 1000 °C for 30 min. After the reaction, the sample was quenched to room temperature, decompressed, and extracted from the platinum capsule to obtain the HP-LGON sample.

## Supplementary Material



## Notes

Experimental methods, atomic positions, occupancies, and atomic displacement parameters; refined parameters of HP-LGON and AP-LGON (Table S1, S2); HP-LGON PLE spectra in energy scale (Figure S1a) and low temperature (Figure S1b); temperature-dependent decay profiles of Ni^2+^ (Figure S2a); pressure-dependent decay profiles of Ni^2+^ luminescence (Figure S2b); calculated average decay times in HP-LGON (Figure S2c); the acquired values of fitting parameters for the temperature-dependent Ni^2+^ luminescence (Table S3); comparison of luminescence and thermal properties with previously reported Ni^2+^doped phosphors (Table S4) (PDF)
