# Supplementary material for: High-Pressure Engineering for the Design of Ni2+-Based Phosphors
Source: ACS Appl Mater Interfaces. 2026 Jun 1;18(22):31499–508. doi: 10.1021/acsami.6c05592 (PMC13308883; doi:10.1021/acsami.6c05592)
Supplement: Supplementary file 1 [file am6c05592_si_001.pdf]

**Supporting Information**

# High-Pressure Engineering for the Design of $Ni^{2+}$-Based Phosphors

*Mikołaj Kamiński[1,*], Yi-Ting Tsai[2], En-Pei Liu[3,4], Wei-Tin Chen[4,5], Mu-Huai Fang[2,*], and Sebastian Mahlik[1]*
*[1]Institute of Experimental Physics, Faculty of Mathematics, Physics and Informatics, University of Gdansk, Wita Stwosza 57, 80-308 Gdansk, Poland*
*[2]Research Center for Applied Sciences, Academia Sinica, Taipei 10608, Taiwan*
*[3]Department of Physics, National Taiwan University, Taipei 10617, Taiwan*
*[4]Center for Condensed Matter Sciences and the Center of Atomic Initiative for New Materials National Taiwan University, Taipei 10617, Taiwan*
*[5]Taiwan Consortium of Emergent Crystalline Materials, National Science and Technology Council, Taipei 10622, Taiwan*
*e-mail: mikolaj.kaminski@ug.edu.pl*
*e-mail:fangmuhai@gate.sinica.edu.tw*

## Experimental

*Synchrotron X-ray Diffraction and Structural Analysis:* Synchrotron powder X-ray diffraction (PXRD) measurements were performed at the National Synchrotron Radiation Research Center (NSRRC), Taiwan, using the TPS BL19A1 beamline. The incident X-ray energy was 16 keV, corresponding to a wavelength of 0.77489 Å. Rietveld refinements of the synchrotron PXRD patterns were carried out using the Total Pattern Analysis Solutions (TOPAS 6.0) software package.

*X-ray Absorption Spectroscopy:* X-ray absorption measurements were conducted at the TPS BL44A1 beamline of NSRRC to investigate the local electronic and structural environment of Ni ions. Ni *K*-edge X-ray absorption near-edge structure (XANES) and extended X-ray absorption fine structure (EXAFS) spectra were collected and analyzed.

*Morphological Characterization:* Scanning electron microscopy (SEM) images were acquired using a Thermo Fisher Phenom Pharos microscope. Optical microscopy (OM) images were obtained with a Keyence VHX-7000 digital microscope.

*Steady-State Photoluminescence Spectroscopy:* Photoluminescence excitation (PLE) and photoluminescence (PL) spectra were recorded using a custom-built optical setup. An EQ99X laser-driven xenon light source (Energetiq), coupled to a home-built grating monochromator covering the 250–1000 nm range, was employed as the excitation source. Emission was

collected using an Andor SR-500i-D1 spectrometer equipped with a CCD camera (DU490A-1.7), operating over the 600–1700 nm spectral range.

*Time-Resolved Photoluminescence Measurements:* Photoluminescence decay profiles were measured using a custom-designed time-resolved setup comprising a SIGLENT SDG2082X function generator to produce square-wave excitation pulses with adjustable repetition rates, a RIGOL HDO4204 200 MHz digital oscilloscope, and an APD110C/M avalanche photodetector sensitive in the 900–1700 nm range. For temperature- and pressure-dependent luminescence measurements, a 420 nm light-emitting diode and an 85 mW 658 nm diode laser were used as excitation sources, respectively.

*Temperature-Dependent Luminescence Measurements:* Temperature-dependent photoluminescence measurements were performed using a Linkam THMS600 temperature stage and an LNP95 liquid nitrogen cooling system, enabling precise temperature control from 77 to 600 K.

*High-Pressure Luminescence Measurements:* High-pressure luminescence measurements were performed using a screw-driven Merrill–Bassett–type diamond anvil cell (DAC) equipped with 0.6 mm culet diamonds. The Inconel gasket was pre-indented to approximately 0.08 mm, and a 0.22 mm-diameter hole was drilled at the center to form the sample chamber. Pressure was calibrated using the ruby fluorescence method, and polydimethylsiloxane (PDMS) oil was employed as the pressure-transmitting medium.[1]

## Results

### *Structural properties*

**Table S1**. The detailed information regarding atomic positions, atomic displacement parameters, occupancy, and refinement parameters of HP-LGON.

| HP-LGO | | | | | | |
|---|---|---|---|---|---|---|
| **Site** | **Np** | **x** | **y** | **z** | **Occ** | **Beq** |
| **Li1** | **3** | **0.00000** | **0.00000** | **0.00000** | **1** | **0.02(1)** |
| **Ga1** | **3** | **0.00000** | **0.00000** | **0.00000** | **1** | **0.12(2)** |
| **O1** | **6** | **0.00000** | **0.00000** | **0.2401(1)** | **1** | **0.01(3)** |
| ***a*** **= 2.91276(1)Å,** ***c*** **= 14.4690(2) Å,** ***V*** **= 106.311(2)Å$^3$,** ***Rp*** **= 1.58%,** ***Rwp*** **= 2.33%, GOF = 5.54** | | | | | | |

**Table S2.** The detailed information regarding atomic positions, atomic displacement parameters, occupancy, and refinement parameters of AP-LGON.

| AP-LGO | | | | | | |
|---|---|---|---|---|---|---|
| Site | Np | x | y | z | Occ | Beq |
| Li1 | 4 | 0.417(1) | 0.136(3) | 0.493(8) | 1 | 0.02(1) |
| Ga1 | 4 | 0.08283(1) | 0.1273(2) | 0.0000 | 1 | 0.12(2) |
| O1 | 4 | 0.4070(6) | 0.1431(9) | 0.9000(6) | 1 | 0.01(3) |
| O2 | 4 | 0.0632(6) | 0.110(1) | 0.3690(7) | 1 | 0.01(3) |
| *a* = 5.406672(8)Å, *b* = 6.37874(1)Å, *c* = 5.012841(8)Å, *V* = 172.8816(5)Å$^3$, *Rp* = 2.20%, *Rwp* = 2.88%, GOF = 2.75 | | | | | | |

## *Luminescence properties*

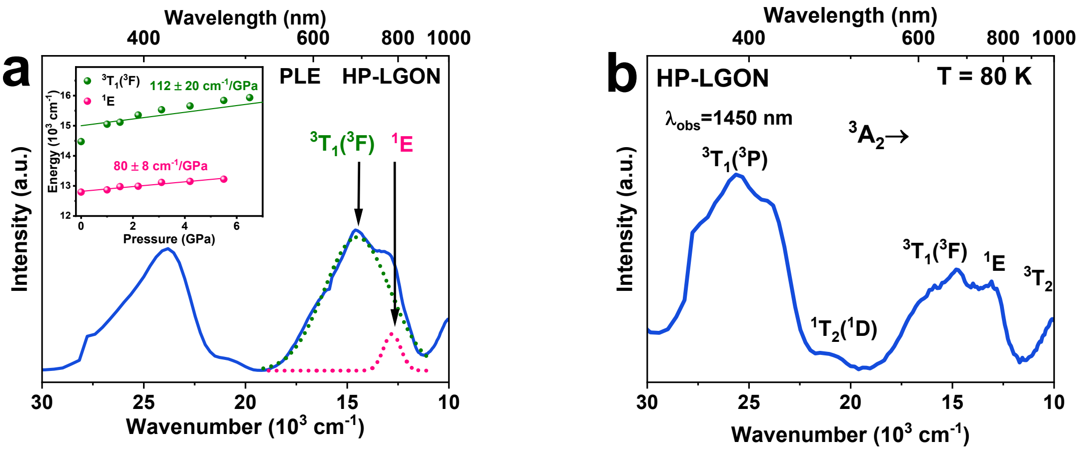


**Figure S1.** (a) HP-LGON PLE spectrum presented on an energy scale, with the pressure-dependent shifts of the Gaussian components shown in the inset. (b) The low temperature (80 K) PLE spectrum of $Ni^{2+}$ emission in HP-LGON .

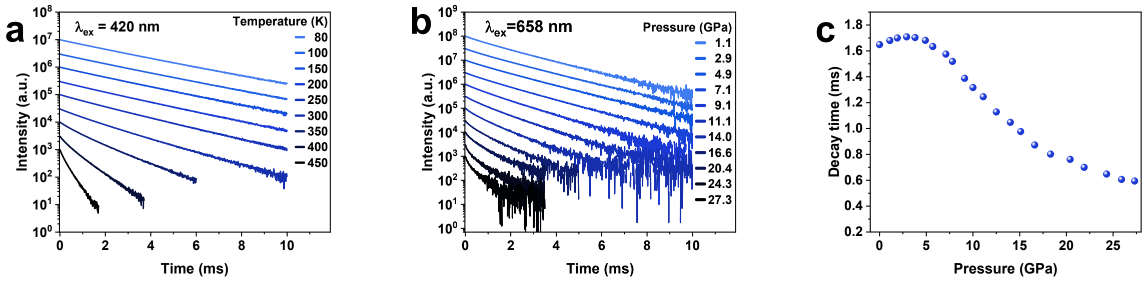


**Figure S2.** (a) Temperature – dependent decay profiles of $Ni^{2+}$, pressure - dependent (b) decay profiles of $Ni^{2+}$ luminescence, (c) calculated average decay times in HP-LGON

**Table S3.** The acquired values of fitting parameters for the temperature-dependent $Ni^{2+}$ luminescence in HP-LGON.

| $A$ | $E_A$ (eq 5) | $\tau_0$ | $\tau$ at 300 K | $p_{nr}$ | $E_A$ (eq 6) |
|---|---|---|---|---|---|
| 985 ± 340 | 1450 ± 75 $cm^{-1}$ | 2.42 ± 0.01 ms | 1.65 ± 0.01 ms | 140 ± 55 $ms^{-1}$ | 1350 ± 100 $cm^{-1}$ |

**Table S4.** Comparison of the luminescence properties of $Ni^{2+}$-activated phosphors reported in the literature, including emission maxima ($\lambda_{max}$), FWHM, activation energy of thermal quenching ($E_A$), and the temperature at which the emission intensity drops to 50% ($T_{50}$).

| Phosphor | $\lambda_{max}$ (nm) | *FWHM* (nm) | $E_A$ ($cm^{-1}$) | $T_{50}$ (K) |
|---|---|---|---|---|
| $SrAlTaO_6$ [2] | 1160 | 180 | 1770 | >430 |
| $LiAl_5O_8$:$Ni^{2+}$ [3] | 1180 | 190 | 3700 | 500 |
| $MgGa_2O_4$:$Cr^{3+}$, $Ni^{2+}$ [4] | 1260 | 224 | 2060 | 420 |
| $LiGa_5O_8$:$Ni^{2+}$ [3] | 1280 | 206 | 4300 | 480 |
| $ZnGa_2O_4$:$Cr^{3+}$,$Ni^{2+}$ [5] | 1285 | 149 | 1330 | 260 |
| MgO:$Cr^{3+}$, $Ni^{2+}$ [6] | 1335 | 259 | - | >500 |
| $Y_2MgTiO_6$: $Cr^{3+}$, $Ni^{2+}$ [7] | 1350 | 235 | 1130 | 375 |
| $La_3Ga_5GeO_{14}$:$Ni^{2+}$ [8] | 1422 | 340 | 1100 | 315 |
| $Y_3Al_2Ga_3O_{12}$-Ni-Zr-$H_3BO_3$ [9] | 1450 | 293 | 1695 | >500 |
| $Mg_{1.88}Ga_{0.14}Sn_{0.9}O_4$:$Cr^{3+}$,$Ni^{2+}$ [10] | 1470 | 295 | - | 230 |
| α-$LiGaO_2$:$Ni^{2+}$ [this work] | 1500 | 278 | 1400 | 300 |
| $NaY_2Ga_2InGe_2O_{12}$:$Cr^{3+}$,$Ni^{2+}$ [11] | 1550 | 313.8 | - | 375 |

## References


(1) Mahlik, S. High-Pressure Study of Phosphors Emission. In *Phosphor Handbook*; CRC Press, 2022.

(2) Zhu, F.; Gao, Y.; Qiu, J. $Sr_2AlTaO_6$: $Ni^{2+}$ Phosphors with Excellent IQE and Thermal Stability as NIR-II Source for Night Vision, Nonvisual Detection, and Far-Field Imaging. *Chemical Engineering Journal* **2025**, *505*, 159559. https://doi.org/10.1016/j.cej.2025.159559.

(3) Kamiński, M.; Tsai, Y.-T.; Kuo, Y.-L.; Muñoz, A.; Rodríguez Mendoza, U. R.; Fang, M.-H.; Mahlik, S. Exploring Near-Infrared Luminescence of $Ni^{2+}$ in $Li(Ga,Al)_5O_8$:Spinels via Mechanical and Chemical Pressure. *Chem. Mater.* **2026**, *38* (2), 910–918. https://doi.org/10.1021/acs.chemmater.5c02959.

(4) Chang, C.-Y.; Huang, M.-H.; Chen, K.-C.; Huang, W.-T.; Kamiński, M.; Majewska, N.; Klimczuk, T.; Chen, J.-H.; Cherng, D.-H.; Lu, K.-M.; Pang, W. K.; Peterson, V. K.; Mahlik, S.; Leniec, G.; Liu, R.-S. Ultrahigh Quantum Efficiency Near-Infrared-II Emission Achieved by $Cr^{3+}$ Clusters to $Ni^{2+}$ Energy Transfer. *Chem. Mater.* **2024**, *36* (8), 3941–3948. https://doi.org/10.1021/acs.chemmater.4c00438.

(5) Satpathy, A.; Huang, W.-T.; Chan, M.-H.; Su, T.-Y.; Kamiński, M.; Majewska, N.; Mahlik, S.; Leniec, G.; Kaczmarek, S. M.; Hsiao, M.; Liu, R.-S. Near-Infrared I/II Nanophosphors with $Cr^{3+}/Ni^{2+}$ Energy Transfer for Bioimaging. *Advanced Optical Materials* **2023**, *11* (15), 2300321. https://doi.org/10.1002/adom.202300321.

(6) Liu, B.-M.; Guo, X.-X.; Cao, L.-Y.; Huang, L.; Zou, R.; Zhou, Z.; Wang, J. A High-Efficiency Blue-LED-Excitable NIR-II-Emitting MgO:$Cr^{3+}$,$Ni^{2+}$ Phosphor for Future Broadband Light Source toward Multifunctional NIR Spectroscopy Applications. *Chemical Engineering Journal* **2023**, *452*, 139313. https://doi.org/10.1016/j.cej.2022.139313.

(7) Xu, Z.; Wang, Y.; Wang, X.; Wu, D.; Shang, M. Blue-Light-Excited $Y_2MgTiO_6$:$Ni^{2+}$, $Cr^{3+}$ Phosphors with Ultrabroadband NIR Emission for Multifunctional Applications. *ACS Appl. Mater. Interfaces* **2025**, *17* (29), 42139–42147. https://doi.org/10.1021/acsami.5c09994.

(8) Tan, M.; Gao, Y.; Chen, J.; Lu, X.; Zhu, B.; Huang, L.; Qiu, J. A Nickel-Doped, Lanthanum Gallium Germanate-Based Phosphor as an Ultra-Broadband Short-Wavelength Infrared Region Emitter for Optical Imaging. *Ceramics International* **2024**, *50* (13, Part B), 23685–23693. https://doi.org/10.1016/j.ceramint.2024.04.092.

(9) Yuan, L.; Jin, Y.; Wu, H.; Deng, K.; Qu, B.; Chen, L.; Hu, Y.; Liu, R.-S. $Ni^{2+}$-Doped Garnet Solid-Solution Phosphor-Converted Broadband Shortwave Infrared Light-Emitting Diodes toward Spectroscopy Application. *ACS Appl. Mater. Interfaces* **2022**, *14* (3), 4265–4275. https://doi.org/10.1021/acsami.1c20084.

(10) Chen, K.-C.; Huang, M.-H.; Huang, W.-T.; Kamiński, M.; Cherng, D.-H.; Lu, K.-M.; Leniec, G.; Mahlik, S.; Liu, R.-S. Tuning Shortwave Infrared Emission Wavelengths by Chemical Pressure in $Cr^{3+}$,$Ni^{2+}$ Co-Doped Spinel Phosphors. *Advanced Optical Materials* **2025**, *13* (19), 2500393. https://doi.org/10.1002/adom.202500393.

(11) Xing, X.; Wang, X.; Wang, Y.; Yu, L.; Xu, Z.; Wu, D.; Shang, M. Blue-Light-Excited NIR-I to NIR-III Emitting $NaY_2Ga_2InGe_2O_{12}$:$Ni^{2+}$, $Cr^{3+}$ Phosphor for Multifunctional Applications. *Ceramics International* **2025**, *51* (25, Part B), 45973–45982. https://doi.org/10.1016/j.ceramint.2025.07.311.
